# Mikronährstoffe: Nutzen, Risiken und öffentliche Wahrnehmung

**DOI:** 10.1007/s00103-025-04140-3

**Published:** 2025-10-14

**Authors:** Anke Weißenborn, Suzan Fiack

**Affiliations:** 1 Abt. Lebens- und Futtermittelsicherheit in der Nahrungskette, Bundesinstitut für Risikobewertung, Max-Dohrn-Str. 8–10, 10589 Berlin, Deutschland; 2 Abt. Risikokommunikation, Bundesinstitut für Risikobewertung, Max-Dohrn-Str. 8–10, 10589 Berlin, Deutschland

Vitamin C für das Immunsystem, Magnesium für die Muskeln oder Vitamin D für die Knochen – Mikronährstoffe sind in großer Zahl und unterschiedlichsten Kombinationen als Nahrungsergänzungsmittel (NEM) auf dem Markt. Sie werden als Kapseln, Tabletten oder Tropfen angeboten und oft damit beworben, eine unzureichende Nährstoffaufnahme auszugleichen oder sogar gesundheitliche Vorteile zu erzielen. In Deutschland greift rund ein Drittel der Erwachsenen und zwischen 5 % und 20 % der Kinder täglich oder mehrmals pro Woche zu NEM. Doch ist dies wirklich notwendig? Wann kann eine gezielte Ergänzung sinnvoll sein – und wann bergen NEM Risiken?

Unbestritten ist, dass unser Körper für die Aufrechterhaltung lebenswichtiger Funktionen auf die Aufnahme von Mikronährstoffen, also Vitaminen und Mineralstoffen, sowie von weiteren Nährstoffen, wie essenziellen Aminosäuren und Fettsäuren, über die Nahrung angewiesen ist. Lebensmittel enthalten darüber hinaus eine Vielzahl anderer Stoffe, darunter Ballaststoffe und sekundäre Pflanzenstoffe (z. B. Carotinoide, Flavonoide und Phytoöstrogene). Auch diese sind heutzutage als sogenannte sonstige Stoffe mit ernährungsspezifischer oder physiologischer Wirkung in NEM zu finden und werden gesundheitlich beworben, so zum Beispiel Koffein zur allgemeinen Leistungssteigerung oder Omega-3-Fettsäuren zur Stärkung des Immunsystems.

Grundsätzlich gilt, dass eine ausgewogene, abwechslungsreiche Ernährung den Nährstoffbedarf eines gesunden Menschen nahezu vollständig decken kann. Ausnahmen sind Vitamin D (das bei ausreichender UVB-Bestrahlung endogen gebildet wird) und Jod, deren natürliche Gehalte in Lebensmitteln nicht ausreichen, um die Versorgung der Bevölkerung sicherzustellen. Darüber hinaus zeigen Studiendaten, dass in Deutschland bestimmte weitere Mikronährstoffe – wie Calcium, Eisen oder Folat – von einzelnen Bevölkerungsgruppen nicht immer entsprechend den Zufuhrreferenzwerten der Deutschen Gesellschaft für Ernährung e. V. (DGE) aufgenommen werden. Wenngleich eine unter dem Zufuhrreferenzwert liegende Aufnahme nicht zwangsläufig einen Mangel mit gesundheitlichen Folgen bedeutet, kann die Supplementierung von einzelnen Vitaminen und/oder Mineralstoffen in bestimmten Lebensphasen oder bei spezifischen Bedürfnissen sinnvoll sein – etwa während der Schwangerschaft (Folsäure und Jod), in der Stillzeit (Jod), im Säuglingsalter (Vitamin K und Vitamin D sowie Fluorid bis zum ersten Zahn), bei bestimmten Ernährungsweisen (z. B. Vitamin B12 bei veganer Ernährung) oder im höheren Lebensalter (nach ärztlicher Rücksprache). Für die Mehrheit der gesunden Bevölkerung sind NEM jedoch nicht notwendig, insbesondere nicht solche mit einem breiten Spektrum an Mikronährstoffen und/oder „sonstigen Stoffen“. Auch ist zu beachten, dass NEM rechtlich Lebensmittel – also *keine* Arzneimittel – sind, selbst wenn sie in Form von z. B. Kapseln oder Tabletten angeboten werden und sich zum Teil auch in der Zusammensetzung und Dosis kaum von Arzneimitteln unterscheiden.

Die Beiträge unseres Themenhefts bewegen sich im Spannungsfeld zwischen Nutzen und Risiken von Mikronährstoffen bzw. wissenschaftlichen Erkenntnissen einerseits und unrealistischen Erwartungen gegenüber einer Mikronährstoffsupplementierung anderseits.

Wir starten mit einem einführenden Artikel von *Schulze et al.*, in dem die grundlegenden Unterschiede zwischen Lebensmitteln, NEM und Arzneimitteln beleuchtet werden, vor allem in Hinblick auf ihre rechtlichen Rahmenbedingungen. Dabei werden auch die praktischen Herausforderungen bei der Abgrenzung von Arzneimitteln und Lebensmitteln und in diesem Zusammenhang Begriffe wie „pharmakologische Wirkung“ und „Erheblichkeitsschwelle“ diskutiert.

Ob, wann und für wen NEM tatsächlich sinnvoll sein können und wann sie eher Risiken bergen – diesen Fragen widmet sich der Beitrag von *Bendadani et al.* Darin wird das Risiko einer unzureichenden oder übermäßigen Nährstoffzufuhr über die herkömmliche Ernährung bewertet und daraus der mögliche Nutzen wie auch die Risiken einer Supplementierung abgeleitet. Anschließend wird ein genauerer Blick auf einige der aktuell beliebtesten Substanzen in NEM geworfen: Vitamin D, Koffein und Omega-3-Fettsäuren. Auch wird thematisiert, dass eine Nahrungsergänzung nur unter bestimmten Bedingungen (spezielle Bevölkerungsgruppen und Ernährungsweisen) sinnvoll sein kann – und welche Risiken, etwa durch Überdosierung oder mögliche Wechselwirkungen, z. B. auch mit Medikamenten, auftreten können.

Ein besonderes Augenmerk widmen wir den spezifischen Bedürfnissen von Menschen in bestimmten Lebensphasen. So beleuchten *Brettschneider et al.* die Bedeutung einer adäquaten Mikronährstoffversorgung bei Säuglingen, Kindern und Jugendlichen, während *Tran et al. *auf die physiologischen Besonderheiten von älteren Menschen eingehen. Auch wenn es noch einige Wissenslücken gibt, lässt sich insgesamt feststellen, dass der Bedarf an Mikronährstoffen je nach Lebensphase und Gesundheitszustand stark variieren kann. Für manche kann eine Ergänzung hilfreich sein, für andere Gruppen birgt sie dagegen Risiken. Eine differenzierte Betrachtung zeigt, wie wichtig es ist, Mikronährstoffergänzungen individuell auf die jeweilige Lebenssituation abzustimmen. Dies gilt nicht nur für bestimmte Lebensphasen, sondern auch für besondere Ernährungsweisen.

Die Bedürfnisse einer speziellen Zielgruppe, die aktuell stark im Fokus der NEM-Werbung steht – Sportlerinnen und Sportler –, werden im Beitrag von *Carlsohn* differenziert betrachtet. Dabei wird aufgezeigt, dass Mikronährstoffdefizite häufig nicht aus einer sportartspezifischen Bedarfssteigerung resultieren, sondern eher aus einer restriktiven Ernährung, inadäquater Energiezufuhr oder erhöhten Verlusten.

Die Sicherheit von NEM ist ein zentrales Thema. Denn: Lebensmittel, so auch NEM, die nicht sicher sind, dürfen nicht vermarktet werden. Im Unterschied zu Arzneimitteln wird ihre Zusammensetzung jedoch nicht vorab behördlich geprüft; die Produkte müssen lediglich beim Bundesamt für Verbraucherschutz und Lebensmittelsicherheit (BVL) angezeigt werden. Aufgabe der Überwachungsbehörden der Bundesländer ist es, die Einhaltung der Rechtsvorschriften bei den auf dem Markt angebotenen NEM zu kontrollieren. Die regelmäßigen stichprobenartigen Kontrollen leisten einen wichtigen Beitrag zum Verbraucherschutz; sie zeigen aber auch, dass bei NEM Vorsicht geboten ist. Dies wird in dem Beitrag von *Krüger und Paschke* exemplarisch für Schleswig-Holstein verdeutlicht. Darin wird auch auf die Herausforderungen durch Regelungslücken sowie auf die Problematik der Abgrenzung von NEM zu Arzneimitteln und neuartigen Lebensmitteln eingegangen.

Doch nicht nur die Substanzen und Produkte selbst verdienen eine kritische Betrachtung, sondern auch die Art und Weise der Vermarktung und ihre Wahrnehmung in der Bevölkerung. *Obstfeld und Lohmann* veranschaulichen dies anhand einer Wahrnehmungsstudie des Bundesinstituts für Risikobewertung (BfR) und *Gauch und Smollich* beleuchten, wie die Bevölkerung über gesundheitliche Risiken von NEM informiert wird und welchen Einfluss soziale Medien darauf haben.

In einer Welt, in der NEM allgegenwärtig sind und die Versprechungen für Gesundheit, Wohlbefinden und Leistungsfähigkeit zunehmen, ist es umso wichtiger, zwischen wissenschaftlich fundierten Erkenntnissen und unrealistischen Erwartungen zu unterscheiden. Wir möchten daher mit diesem Themenheft dazu beitragen, die komplexen Zusammenhänge von Nutzen und Risiken von NEM mit Mikronährstoffen und „sonstigen Stoffen“ verständlicher zu machen – und hoffen, dass die Beiträge Orientierung bieten.

In diesem Sinne wünschen wir Ihnen eine spannende und aufschlussreiche Lektüre!

Suzan Fiack und Anke Weißenborn

